# Real-life effectiveness of once-daily single-inhaler triple therapy (FF-UMEC-VI) after switching from dual therapy (ICS-LABA) in patients with symptomatic asthma: trelegy ellipta for real asthma control study

**DOI:** 10.3389/falgy.2025.1537501

**Published:** 2025-03-17

**Authors:** Yoshitomo Kushima, Yasuo Shimizu, Ryo Arai, Kazuyuki Chibana, Yuka Shimizu, Masahiro Amagai, Akihiro Takemasa, Naoya Ikeda, Meitetsu Masawa, Atsushi Kushima, Hiroaki Okutomi, Yusuke Nakamura, Rinna Tei, Yuki Ando, Nana Yazawa, Yuto Goto, Yasuo Haruyama, Tatsuo Yukawa, Seiji Niho

**Affiliations:** 1 Department of Pulmonary Medicine and Clinical Immunology, Dokkyo Medical University, Mibu, Tochigi, Japan; 2 Kushima Internal Medicine Clinic, Moka, Tochigi, Japan; 3 Amagai Internal Medicine Clinic, Tochigi, Tochigi, Japan; 4 Integrated Research Faculty for Advanced Medical Sciences, Dokkyo Medical University, Tochigi, Japan; 5 Yukawa Clinic of Internal Medicine, Utsunomiya, Tochigi, Japan

**Keywords:** asthma, cognition, fluticasone furoate, forced expiratory flow volume, trelegy, triple therapy, umeclidinium, vilanterol

## Abstract

**Introduction:**

A well-designed, protocol-driven randomized controlled trial (RCT) has demonstrated the efficacy of fluticasone furoate-umeclidinium-vilanterol (FF-UMEC-VI) in patients with asthma, but there is a lack of real-world data that can be used to translate the results of the RCT into clinical practice. This study evaluated the efficacy of switching the therapy from inhaled corticosteroid-long-acting β2-agonists (ICS-LABAs) to FF-UMEC-VI at the equivalent corticosteroid dose in a real-world setting.

**Methods:**

A prospective, three-month, open-label, parallel-group, switching therapy trial was performed in patients with symptomatic asthma under routine management. Patients receiving low-to-medium doses of ICS-LABAs were switched to FF-UMEC-VI (100–62.5–25 µg, once daily) (T100 group), and patients receiving a high dose of ICS-LABAs were switched to FF-UMEC-VI (200–62.5–25 µg, once daily) (T200 group). The primary outcome was the change from baseline in forced expiratory volume in 1 s (ΔFEV1) at week 12, and the secondary outcomes were the improvement in fractional exhaled nitric oxide (FeNO), the asthma symptoms evaluated using the asthma control test (ACT), and the cough severity evaluated using the visual analog scale (VAS).

**Results:**

Thirty-five patients were switched to T100, and thirty patients were switched to T200. The ΔFEV1 was improved by more than 100 ml at 8 weeks after switching in both groups (T100, 110.4 ± 39.8 ml; T200, 117.1 ± 39.8 ml) (*p* < 0.05) but slightly decreased at 12 weeks. ACT also improved by more than 3 points at 8 weeks after switching and was maintained to 12 weeks in both groups (*p* < 0.05). Patients with ACT scores of <20 (i.e., poor control) before switching showed a greater improvement in the symptoms during T100 therapy, and 92% had reached an ACT score of >20 (i.e., good control). FeNO in the T100 group was decreased at 4 weeks (*p* < 0.05). Cough VAS also significantly decreased but did not reach a minimal clinically important difference.

**Conclusions:**

In patients with symptomatic asthma showing insufficient control, an improvement in the asthma symptoms was observed after switching to FF-UMEC-VI at the equivalent corticosteroid dose, accompanied by an improvement in FEV1.

## 1 Introduction

Therapy with inhaled agents is useful in the treatment of asthma because it requires much lower doses than oral medications, has fewer side effects, and can be used in many countries. Even in the era of biotherapy, inhalation therapy still needs to be refined. The goals of asthma management are to achieve good control of symptoms and minimize future risk of asthma-related mortality, exacerbations, persistent airflow limitation, and adverse effects of the treatment (1). When prescribing a drug to a patient with poorly controlled asthma, it is important to understand the expected benefit of the drug and select the appropriate medication.

Although inhaled corticosteroid-long-acting β2-agonist (ICS-LABA) is often used for initial treatment and maintenance therapy, approximately 30%–50% of adult patients with asthma prescribed ICS-LABA were reported to have inadequate symptom control (2, 3).

In the CAPTAIN trial, which studied the efficacy of ICS-long-acting muscarinic antagonist-LABA (ICS-LAMA-LABA) and fluticasone furoate-umeclidinium-vilanterol (FF-UMEC-VI) as single-inhaler triple therapies (SITTs) in poorly controlled patients with asthma, the SITTs improved asthma symptoms and lung function compared with the fluticasone furoate-vilanterol (FF-VI) dual therapy (4). UMEC appears to improve the forced expiratory volume in 1 s (FEV1), independently of blood eosinophils and fractional exhaled nitric oxide (FeNO). In contrast, a high dose of ICS or FF provided greater improvements in lung function and reduced the exacerbations in patients with higher blood eosinophil counts and FeNO (4). In recent years, the treatable traits strategy has become a promising treatment concept to help overcome refractory asthma (5). The results of the CAPTAIN trial indicated that it is important to find treatable traits, such as high blood eosinophil counts and FeNO, in patients with asthma.

Although the study design of the CAPTAIN trial was robust and contributed to guiding effective therapies, the study was conducted as a randomized controlled trial (RCT) using a switching medication method that was somewhat different from a pragmatic clinical setting. In the CAPTAIN trial, patients with asthma receiving medium- or high-dose ICS-LABA were switched to medium-dose ICS-LABA (250–50 µg of fluticasone propionate-salmeterol, FP-SM), then to medium-dose ICS-LABA (100–25 µg of FF-VI), and finally randomized to the medium- or high-dose FF-UMEC-VI group (4). This pre-licensed RCT would not usually be followed or performed in practice. Global initiative for asthma (GINA) 2022–2024 states that adding LAMA to medium or high dose ICS-LABA modestly improves lung function (6–8), but with no clinically important change in quality of life and symptoms. GINA's step 5 treatment options include LAMA add-on, high-dose ICS-LABA, and refer for phenotypic assessment ± biologic therapy. What these indicate are a requirement to take into consideration the phenotype of patients with asthma requiring step 5 therapy. On the other hand, it is difficult for the general clinician to determine from these descriptions how to use triple therapy in patients who are poorly controlled despite ICS-LABA therapy. GINA was built on the evidence of the RCT. Therefore, practical real-world validation is needed for an additional understanding of GINA for triple therapy. Although there have been sub-analyses of RCTs or retrospective analyses that collected medical record data about the effect of switching from ICS-LABA or combination of ICS and LABA to Trelegy® Elipta® (9–12), no prospective studies have examined the clinical benefit of it. Overall, further studies are needed to clarify the efficacy of FF-UMEC-VI in treating asthma symptoms and determine the biomarker changes in real-world clinical practice.

Therefore, we conducted a prospective study to evaluate the clinical benefit of switching from ICS-LABA to FF-UMEC-VI (Trelegy® Elipta®) in adult patients with asthma with residual asthma symptoms in real-world clinical practice by measuring asthma symptoms, lung function, and biomarkers.

### 1.1 Methods

A prospective, three-month, open-label, parallel-group, switching therapy trial was performed in symptomatic patients with asthma at four healthcare facilities, including one university hospital and three clinics in Japan, to assess the effectiveness of switching from a dual therapy composed of ICS-LABA to FF-UMEC-VI.

**Figure 1:**
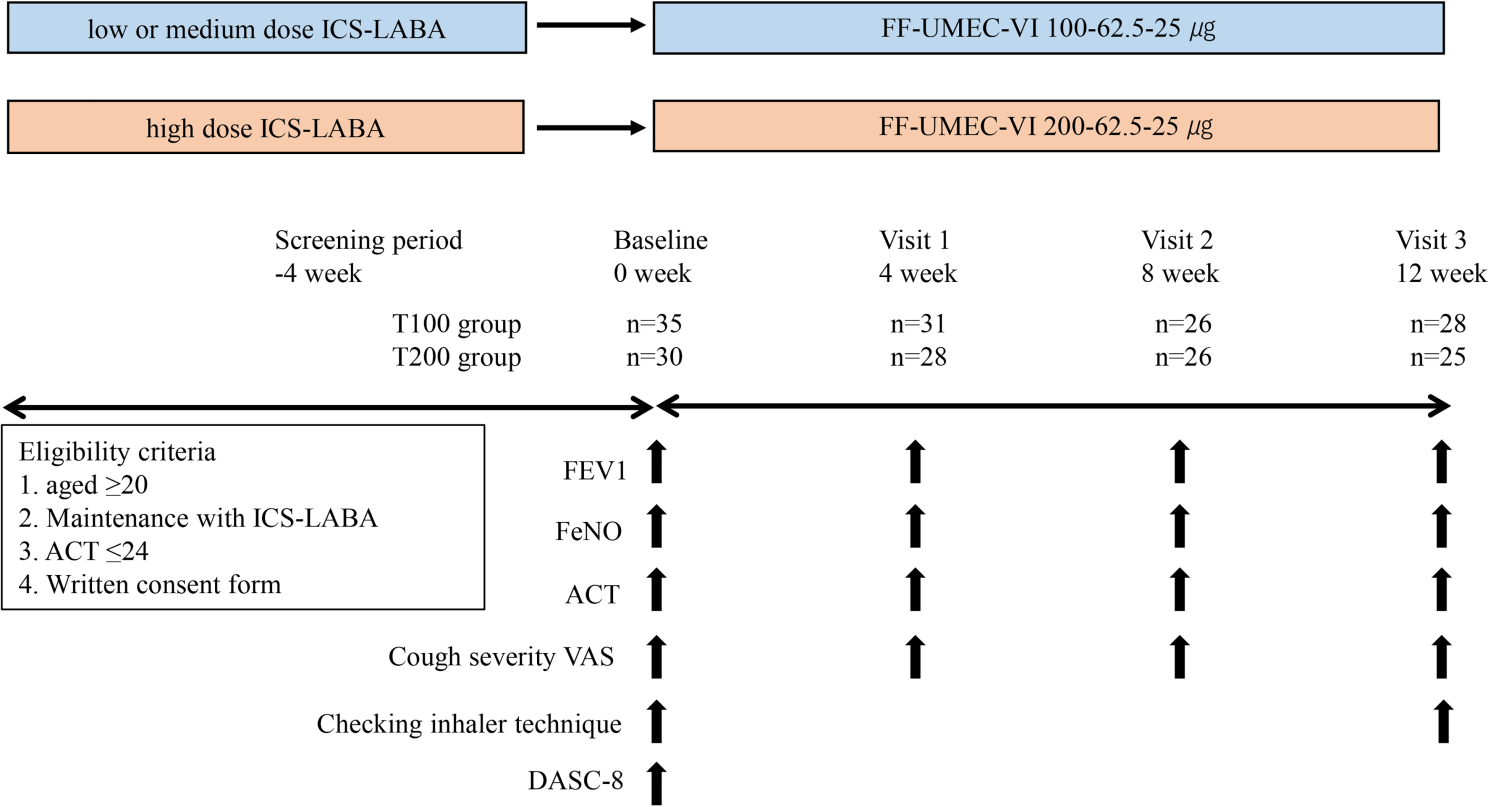
Study design. Patients with asthma receiving ICS-LABA were enrolled. At baseline, ICS-LABA was switched to FF-UMEC-VI using the equivalent ICS dose. Patients visited the hospital every 4 weeks until week 12 (baseline, visit 1, visit 2, visit 3), and various parameters were evaluated every visit, including FEV1, FeNO, ACT, and cough VAS. Checking the inhalation technique for the Elipta® device was performed on the day of baseline and visit 3. DASC-8 was measured on the day of baseline. ICS-LABA, inhaled corticosteroid/long-acting β2-agonist; FF-UMEC-VI, fluticasone furoate-umeclidinium-vilanterol; FEV1, forced expiratory volume in 1 s; FeNO, fraction of exhaled nitric oxide; ACT, asthma control test; VAS, visual analog scale; DASC-8, assessment sheet for cognition and daily function-8 items.

Patients using a low-to-medium dose of ICS (1) were switched to FF-UMEC-VI (100–62.5–25 µg, once daily) (Trelegy® Elipta® 100, T100), referred to as the medium-dose T100 group, and patients using a high dose of ICS were switched to FF-UMEC-VI (200–62.5–25 µg, once daily) (Trelegy® Elipta® 200, T200), referred to as the high-dose T200 group. The screening visit occurred 4 weeks prior to switching therapy, and patients were switched to T100 or T200 at baseline (week 0). Parameters were measured every 4 weeks from baseline to visit 3 (week 12) (Figure 1). The primary outcome was the change from baseline in forced expiratory volume in 1 s (ΔFEV1) at week 12. The minimal clinically important difference (MCID) of ΔFEV1 was defined as 100 ml (13). Spirometry was performed before noon, and T100 or T200 therapy was not stopped on the day of measurement. FEV1 was measured using the FUDAC-7 system at the university hospital (Fukuda Denshi, Co., Ltd., Japan), AUTOSPIRO SYTEM-7W (Minato Medical Science, Co., Ltd.,) at the Yukawa Clinic of Internal Medicine, SP-370COPDHAIPer (Fukuda Denshi, Co., Ltd., Japan) at the Amagai Internal Medicine Clinic, and MICROSPIRO HI-302 (Nihon Kohden, Co., Ltd., Japan) at the Kushima Internal Medicine Clinic. Secondary outcomes were the improvement in asthma symptoms evaluated using the asthma control test (ACT) (QualityMetri, Inc., Lincoln, RI, USA) and the change in FeNO. The ACT score was used to classify patients as follows: ACT < 20 represented poor control, ACT ≥ 20 and ≤24 represented good control, and ACT = 25 represented complete control. The MCID of the ACT score was defined as a shift of 3 points or more (14). FeNO was measured using a SIEVERS NO analyzer (MODEL-280i NOA, Sievers Instruments, Boulder, CO, USA) or NIOX VERO (CHEST CO., Ltd., Japan) at the university hospital, and using NIOX VERO at the three clinics. The MCID of FeNO was defined as a reduction of more than 10 ppb (15). The degree of cough symptoms was measured using the visual analog scale (VAS), and the MCID was defined as a change of 15 mm (16). Instruction regarding the necessary inhalation technique for using the Elipta® device was provided on the day of baseline and visit 3. The protocol for the instructions consisted of 14 items, and the pharmacist checked the items in which a patient made a mistake. The total number of items in which the patient made a mistake was recorded. To investigate the association between the number of mistakes and impaired cognitive function, a dementia assessment sheet for community-based integrated care system 8-items (DASC-8) was used at the day of baseline. DASC-8 was used as the screening tool for cognition and components of comprehensive geriatric assessment. A score of 11 or higher is considered mild dementia and a score of 17 or higher is considered moderate dementia (17).

### 1.2 Patients

Eligibility criteria included patients with asthma aged ≥20 years, use of ICS-LABA for at least 3 months prior to enrollment in this study, symptomatic asthma (ACT ≤ 24), and written informed consent to participate in the study. Exclusion criteria were an age of <20 years, ACT = 25, intercurrent infection, concomitant or pre-existing lung cancer and still undergoing treatment, and known or suspected allergy to FF-VI. To minimize the inclusion of patients with chronic obstructive pulmonary disease (COPD) complications, patients with a smoking history of more than 10 pack-years were also excluded. During the study period, ICS, LABA, and LAMA were not changed, and other concomitant medications were allowed to continue without changing the dosage. Short-acting beta-agonists (SABA), oral corticosteroids, injectable steroids and/or theophylline, and nebulized inhalation of beta-agonists were allowed as relief medications for symptoms of asthma exacerbation. This study was conducted following the Declaration of Helsinki. All patients gave written informed consent before enrollment. This study was approved by the Human Research Committee at Dokkyo Medical University and was registered as clinical trial number R-43-5J and registered with the University Hospital Medical Information Network (UMIN000043481, 2021/3/1, Trelegy Elipta for Real Asthma Control Study).

### 1.3 Safety

Safety endpoints included severe adverse events and the percentage of patients who stopped medication during the study period. If a change to another drug of ICS, LAMA, or LABA other than FF-UMEC-VI was required, or FEV1 dropped 30% compared with the previous visit, the patients were considered dropout cases.

### 1.4 Statistical analysis

A sample size calculation showed that 22 patients in each group were required to provide 80% power with a two-sided *α* risk of 5% to detect a difference of 90 ml or over in FEV1, as previously reported (18). Considering that this study was a real-world multi-center collaboration and the study period was 12 weeks, it was estimated that approximately 50% of the patients who enrolled but dropped out during the screening period after enrollment withdrew during the study or were ineligible for the study (19, 20). This study was designed to allow up to 35 patients in each group. Results are expressed as the mean and SD or median interquartile range (IQR).

**Figure 2:**
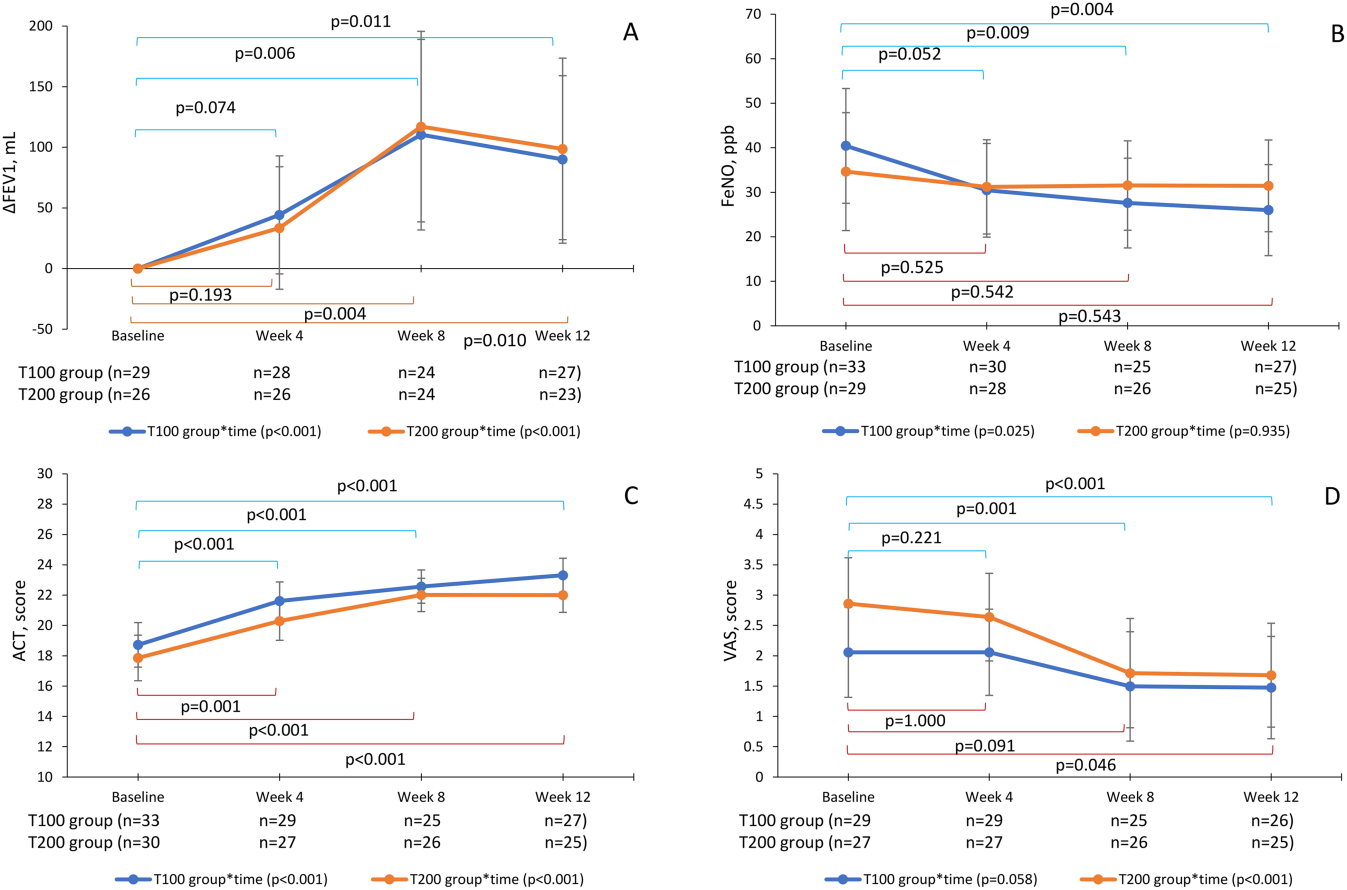
Changes in the parameters by groups and times with GLMMs. ΔFEV1 **(A)**, FeNO **(B)**, ACT **(C)**, and cough VAS **(D)** GLMMs were used to evaluate changes in parameters at baseline, week 4, week 8, and week 12 in the T100 and T200 groups. The T100 group (blue line) switched from ICS-LABA to 100-62.5-25 µg of FF-UMEC-VI, and the T200 group (orange line) switched to 200-62.5-25 µg of FF-UMEC-VI. Points represent the estimated means adjusted by age, sex, body mass index, onset time, and smoking condition. The bars represent the 95% CI, *p*-values are for repeated measurements within a group, and the Least significant difference (LSD) method was used for time comparisons. GLMM, generalized linear mixed model; ICS-LABA, inhaled corticosteroid/long-acting β2-agonist; FF-UMEC-VI, fluticasone furoate-umeclidinium-vilanterol; FEV1, forced expiratory volume in 1 s; FeNO, fraction of exhaled nitric oxide; ACT, asthma control test; VAS, visual analog scale; CI, confidence interval.

Categorical variables were expressed as numbers and percentages, and continuous variables were expressed as the mean and SD between the T100 and T200 groups. Due to small sample size and nonparametric data, the Kruskal–Wallis test was performed for ΔFEV1 (ml), FeNO (ppb), ACT, and VAS scores between the T100 and T200 groups at baseline, week 4, week 8, and week 12. Generalized linear mixed models (GLMM) were used to examine changes in parameters at the four time points, excluding the missing values at each time. Figures 2A–D shows the estimated mean and 95% CI for each parameter at each time, adjusted by age, sex, body mass index, onset time, and smoking condition. Changes over time between groups were evaluated by repeated measures, and comparisons of the parameters between the baseline and each time point were evaluated using the Least significant difference (LSD) method. We compared the baseline and the inhalation technique errors at week 12 (times/patient) using the Wilcoxon signed-rank test Two-tailed *p*-values of <0.05 were considered significant. IBM SPSS software version 29.0 (IBM SPSS, Inc., Tokyo, Japan) and GraphPad Prism 10.2.0 (GraphPad Software, Boston, MA, USA) were used for statistical analyses.

## 2 Results

### 2.1 Background and baseline characteristics of the study population

A total of 65 patients were enrolled from four medical facilities between March 2021 and July 2023. The clinical characteristics of the enrolled patients are shown in Table 1. Thirty-five patients were switched to an FF-UMEC-VI dose of 100–62.5–25 µg (low- or medium-dose ICS, T100 group), and thirty patients were switched to an FF-UMEC-VI dose of 200–62.5–25 µg (high-dose ICS, T200 group). There were no patients who dropped out during the screening period. Females were predominant in both groups, and FF-VI and budesonide-formoterol (BUD-FM) were the most commonly used ICS-LABAs before switching to FF-UMEC-VI. The number of patients with oral corticosteroid (OCS) use was small in both the T100 and T200 groups. The mean ACT score at baseline was 18.7 ± 3.7 in the T100 group and 17.9 ± 4.1 in the T200 group, indicating poor control, and both groups had a long average history of asthma, approximately 20 years. FEV1 was 2,322 ± 700 ml in the T100 group and 2,308 ± 645 ml in the T200 group, and %FEV1 was above 80% in both groups, but the forced expiratory volume/forced vital capacity (FEV1/FVC) was below 80%. DASC-8 scores averaged less than 10 in both groups, with only 1 patient in the T100 group and 2 patients in the T200 group exhibiting scores of 11 or more, suggesting cognitive impairment.

**Table 1:** Characteristics of the patients.

Baseline demographics	100–62.5–25 µg of FF-UMEC-VI	200–62.5–25 µg of FF-UMEC-VI
	(low- or medium-dose ICS, T100 group)	(hige-dose ICS, T200 group)
	(*n* = 35)	(*n* = 30)
Age, years	59.1 (14.5)	54.0 (13.7)
Male	11 (31%)	9 (30%)
Female	24 (69%)	21 (70%)
Maintenance ICS-LABA	FF-VI 14 (40%)	FF-VI 16 (53%)
	BUD-FM 16 (46%)	BUD-FM 11 (37%)
	FP-FM 3 (8%)	FP-FM 1 (3%)
	FP-SM 1 (3%)	FP-SM 2 (7%)
	CIC-SM 1 (3%)	
OCS mg/day, number of patients	6 (0.0), 1	2.4 (2.2), 3
Disease duration, years	19.0 (16.7)	20.2 (16.1)
Former smoker	12 (34%)	9 (30%)
ACT score	18.7 (3.7)	17.9 (4.1)
Cough severity VAS	3.5 (2.3)	3.7 (2.5)
FeNO, ppb	41.2 (43.7)	36.2 (36.3)
FEV1 (ml), *Z-score (median, IQR)*	2,322 (700), *0.004* (*−0.993, 0.764*)	2,308 (645), *−0.126 *(*−0.878, 0.798*)
%FEV1 (predicted)	93.8 (24.8)	88.0 (19.8)
FEV1/FVC (%), *Z-score (median, IQR)*	77.7 (11.2), *0.095 *(*−0.604, 0.785*)	74.1 (12.2), *0.216, *(*−0.885, 0.731*)
DASC-8	8.9 (1.5)	8.8 (1.2)

Data are expressed as *n* (%), mean and SD or median (IRQ). FF-UMEC-VI, fluticasone furoate-umeclidinium-vilanterol; FF-VI, fluticasone furoate-vilanterol; BUD-FM, budesonide-formoterol; FP-FM, fluticasone propionate- formoterol; FP-SM, fluticasone propionate-salmeterol; OCS, oral corticosteroids; FEV1, forced expiratory volume in 1 s; FeNO, fraction of exhaled nitric oxide; ACT, asthma control test; VAS, visual analog scale; DASC-8, assessment sheet for cognition and daily function-8 items.

### 2.2 Findings from subsequent visits

Univariate analysis was performed to examine the changes in parameters at baseline, week 4, week 8, and week 12 in the T100 and T200 groups. ΔFEV1, ACT, and cough VAS changed after switching to FF-UMEC-VI in the two groups (*p* < 0.05), but FeNO was not significantly different in either group (Table 2).

**Table 2:** Changes in parameters from baseline after switching to FF-UMEC-VI.

Parameters	Baseline	Week 4	Week 8	Week 12	*p* value^a^
T100					
ΔFEV1, ml	0	44.1 (112.8)	106.8 (201.2)	90.0 (185.1)	0.002
*n*	35	29	25	28	
FeNO, ppb	41.2 (43.7)	31.5 (26.4)	29.5 (20.2)	27.5 (18.1)	0.88
*n*	35	31	26	28	
ACT, score	18.7 (3.7)	21.6 (2.7)	22.6 (2.7)	23.4 (2.1)	<0.001
*n*	35	30	26	28	
Cough VAS score	3.6 (2.3)	1.9 (1.8)	1.5 (1.3)	1.3 (1.2)	<0.001
*n*	35	30	26	27	
T200					
ΔFEV1, ml	0	33.5 (145.8)	117.1 (185.2)	106.5 (174.1)	<0.001
*n*	30	26	24	23	
FeNO, ppb	36.2 (36.3)	31.3 (29.8)	32.2 (31.5)	31.8 (30.1)	0.972
*n*	29	28	26	25	
ACT, score	17.9 (4.1)	20.3 (4.4)	21.9 (3.3)	21.8 (3.9)	<0.001
*n*	30	27	26	25	
Cough VAS score	3.9 (2.5)	2.5 (1.9)	1.6 (1.6)	1.6 (1.7)	<0.001
*n*	30	27	26	25	

Data are mean (SD).

a The Kruskal–Wallis test was used but the missing data were excluded.

GLMMs were also used to evaluate changes in parameters at baseline, week 4, week 8, and week 12 in the T100 and T200 groups. ΔFEV1 was increased at week 4 and reached the MCID of 100 ml at week 8 in both groups [T100, 110.4 ± 39.8 ml, 95% CI (31.8–189.0); T200, 117.1 ± 39.8 ml, 95% CI (38.5–195.7)] (*p* < 0.05), but slightly decreased at week 12 [T100, 90.0 ± 35.0 ml, 95% CI (20.9–159.1); T200, 98.7 ± 23.8 ml, 95% CI (23.9–173.5)] (*p* < 0.05) (Figure 2A). The primary endpoint of ΔFEV1 at week 12 was increased (*p* < 0.001) but slightly less than the MCID of 100 ml.

FeNO in the T100 group was reduced at week 4 [30.4 ± 5.3 ppb, 95% CI (19.9–41.0)], reaching the MCID of 10 ppb (*p* < 0.05), and then continued to improve until week 12 [26.0 ± 5.2 ppb, 95% CI (15.8–36.2)] (*p* < 0.05). However, in the T200 group, FeNO was not significantly reduced from baseline [34.7 ± 6.7 ppb, 95% CI (21.4–47.9)] during the 12 weeks [31.5 ± 5.2 ppb, 95% CI (21.1–41.6)] (Figure 2B). There was no correlation between baseline FeNO values and ΔFEV1 (data not shown).

ACT scores also improved and reached the MCID of 3 points at 8 weeks in both groups [T100, 22.6 ± 0.6, 95% CI (21.5–23.7); T200, 22.0 ± 0.6, 95% CI (20.9–23.1)] (*p* < 0.05) and were maintained until week 12 (*p* < 0.05) (Figure 2C). There was no correlation between baseline FeNO and a change in ACT score (data not shown).

The baseline of cough VAS was 2.9 ± 0.4 [95% CI (2.1–3.6)] in the T200 group and 2.1 ± 0.4 [95% CI (1.3–2.8)] in the medium group, which significantly decreased by week 8 [T100, 1.5 ± 0.5, 95% CI (0.59–2.40); T200, 1.7 ± 0.5, 95% CI (0.81–2.61)] (*p* < 0.05) and was maintained until week 12 [T100, 1.5 ± 0.4, 95% CI (0.63–2.31), T200, 1.7 ± 0.4, 95% CI (0.82–2.54)] (*p* < 0.05). Cough VAS in both groups were improved but did not reach the MCID, a decrease of 1.5 (Figure 2D).

**Figure 3:**
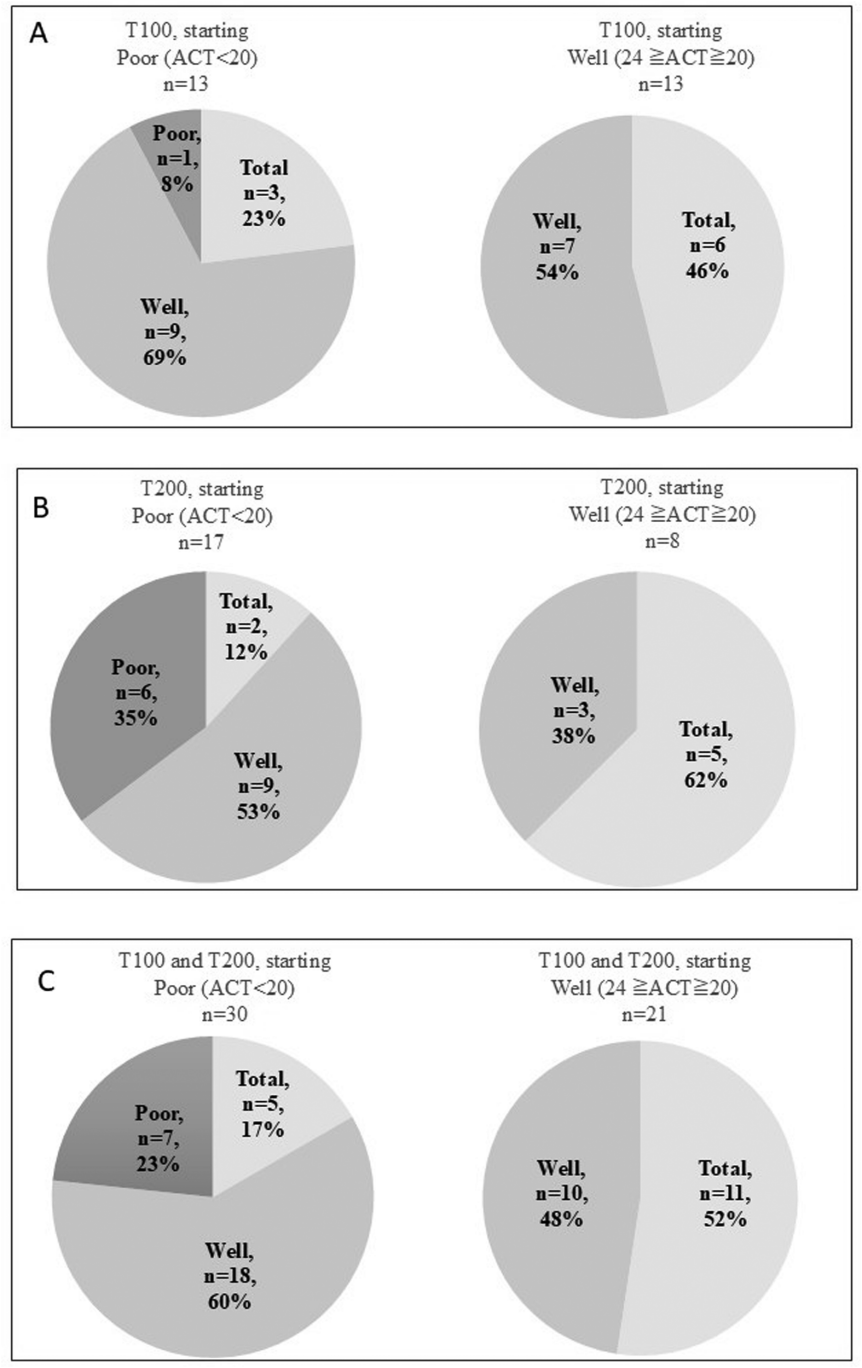
ACT scores and the changes in patients after switching to 100-62.5-25 µg of FF-UMEC-VI (T100 group) **(A)**, 200-62.5-25 µg of FF-UMEC-VI (T200 group) **(B)** Combined T100 and T200 group data for ACT scores before and after switching to FF-UMEC-VI **(C)** Patients with ACT scores of <20 before starting FF-UMEC-VI were defined as having poor control, patients with ACT scores of ≥20 and ≤24 before starting FF-UMEC-VI were defined as having good control, and a score of 25 was defined as total control. FF-UMEC-VI, fluticasone furoate-umeclidinium-vilanterol; ACT, asthma control test.

The ratios of patients in the T100 group with a baseline ACT score of <20 (poorly controlled asthma, *n* = 13) displayed marked improvements at week 12. Specifically, 92% of them became >20 (good control), and 23% of them reached ACT = 25 (total control). Patients in the T100 group with a baseline ACT score of ≥20 (*n* = 13) also showed improvements in asthma symptoms at week 12, and 46% of them reached total control (Figure 3A). Patients in the T200 group with a baseline ACT score of <20 (*n* = 17) displayed marked improvements at week 12. Notably, 65% of them showed a score of >20, and 12% of them reached total control, but 35% remained under poor control. Patients in the T200 group with a baseline ACT score of ≥20 (*n* = 8) also demonstrated improvements in asthma symptoms at week 12, and 62% of them reached total control (Figure 3B). Ratios combing patients in both groups with a baseline ACT score of <20 (*n* = 30) displayed marked improvements at week 12, and 77% of them had a score of >20, where 17% of them reached total control, although 23% remained under poor control. Combing patients in both groups with a baseline ACT score of ≥20 (*n* = 21) also showed improvement at week 12, and 52% of them reached total control (Figure 3C).

**Figure 4:**
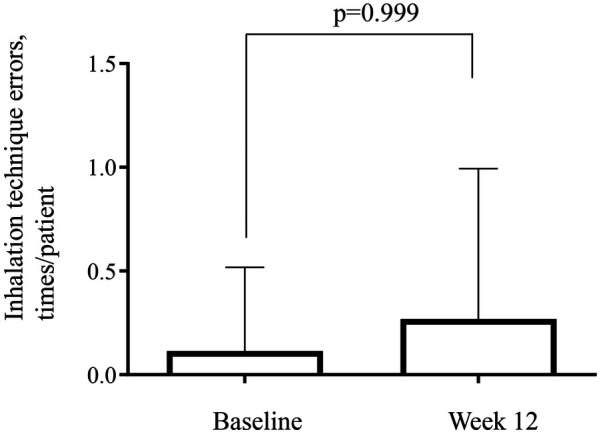
Number of errors for the inhalation technique using the trelegy® elipta® device. Combined data is shown from the T100 (100-62.5-25 µg of FF-UMEC-VI) and T200 (200-62.5-25 µg of FF-UMEC-VI) groups. Inhalation techniques were checked at baseline and on the final visit (week 12). FF-UMEC-VI, fluticasone furoate-umeclidinium-vilanterol.

The combined data for the number of errors made while performing the inhalation technique (times/patient) in the T100 and T200 groups showed values of 0.11/patient at baseline and 0.29/patient at week 12, which did not significantly differ (Figure 4).

The most common adverse event was hoarseness in both groups [2 patients (6%) in the T100 group and 3 patients (10%) in the T200 group]. In the T100 group, bitter taste was mostly reported [3 patients (9%)], followed by pharyngeal discomfort, cough, and dry mouth. Three patients in each of the groups dropped out after starting the study due to adverse drug reactions. The adverse events that occurred in those 6 patients were hoarseness, pharyngeal discomfort, cough, numbness of tongue, and bitter taste. There were no serious adverse events (Table 3).

**Table 3:** Adverse events during study period.

Adverse events	T100 (*n* = 35)	T200 (*n* = 30)
Hoarseness	2 (6%)	3 (10%)
Bitter taste	3 (9%)	1 (3%)
Sputum	0	2 (7%)
Pharyngeal discomfort	1 (3%)	0
Cough on inhalation	1 (3%)	0
Dry mouth	1 (3%)	0
Numbness of tongue	0	1 (3%)
Hiccups	0	1 (3%)
Nasal congestion	0	1 (3%)
Adverse events leading to study treatment discontinuation	3 (9%)	3 (10%)

## 3 Discussion

The present study showed that switching from ICS-LABA to FF-UMEC-VI with the equivalent dose of ICS improved asthma symptoms and FEV1 in patients with residual asthma symptoms despite prior treatment of a low-to-medium or high dose of ICS-LABA. Improvements in FeNO were modest compared with improvements in the ACT score and FEV1, and high-dose FF-UMEC-VI did not show significant reductions in FeNO. There was no correlation between baseline FeNO values and the improvement in asthma symptoms, or baseline FeNO values and the improvement in FEV1. Accordingly, this study showed that when patients with asthma on ICS-LABA therapy have residual asthma symptoms, switching to FF-UMEC-VI contributes to an improvement in asthma symptoms and FEV1, regardless of the FeNO level.

The change in FEV1 reached the MCID after 8 weeks, with a significant difference in medium- and high-dose FF-UMEC-VI, and the improvement was almost maintained until week 12. The CAPTAIN study showed that switching to medium- or high-dose FF-UMEC-VI resulted in FEV1 improvements exceeding the MCID of 100 ml, similar to the present results (4). We speculated that airway reversibility to LAMA remained in patients receiving either a low-to-medium or high-dose of ICS-LABA, despite a low baseline level of FEV1/FVC (approximately 70%), which is almost considered obstructive ventilatory impairment. Thus, switching from medium- or high-dose ICS-LABA to the equivalent ICS dose of FF-UMEC-VI may improve FEV1. The CAPTAIN study recommended increasing the dose of ICS and adding LAMA based on biomarkers such as eosinophils, FeNO, and pulmonary functions (4). Considering that lower FEV1 is associated with disease progression and prognosis in patients with asthma (21, 22), FF-UMEC-VI therapy may reduce these risks in patients with asthma.

The findings suggest that airway reversibility or airflow limitation is a treatable trait using an appropriate SITT. In particular, FF-UMEC-VI therapy may be effective in patients with low FEV1 and/or airflow reversibility. However, FEV1 measurements by spirometry are not always available. A previous study demonstrated an increase in FEV1 with the addition of LAMA to ICS-LABA, as a triple therapy, and home measurements of morning peak expiratory flow (PEF) and FEV1 using a portable device were useful in determining treatment response (23). Further work is needed to verify whether PEF can replace FEV1 in predicting SITT efficacy.

The effect of FF-UMEC-VI on FeNO reduction was more pronounced in patients receiving a medium dose of FF-UMEC-VI, but less notable at a high dose in the present study. There are several potential reasons for this. First, the baseline FeNO was originally higher in the medium-dose patients than in the high-dose patients. Many of the patients treated with a medium dose of ICS-LABA had residual inflammation. Second, a higher percentage of BUD-FM was prescribed as a pretreatment in the medium-dose patients, so the strong anti-inflammatory effect of FF-VI may contribute to the reduction of FeNO. It has also been shown that an improvement in FeNO is obtainable by switching to FF-VI at the equivalent corticosteroid dose from other ICS-LABAs (20). Therefore, when patients are treated with medium-dose ICS-LABAs with residual type 2 inflammation (i.e., patients receiving inadequate anti-inflammatory therapy), switching to medium-dose FF-UMEC-VI contributes to the reduction in airway inflammation, which can help alleviate asthma symptoms. Thus, the types of ICS-LABA before switching could influence the treatment outcomes. However, when FeNO is suppressed near the upper limit of the normal range, such as in patients treated with a high dose of ICS-LABAs, it can be assumed that the anti-inflammatory effect of FF-VI is approximately saturated, and switching to a high dose of FF-UMEC-VI is not expected to strengthen the anti-inflammatory effect. Overall, in the case of patients with high FeNO, switching to a high dose of FF-UMEC-VI may reduce airway inflammation.

The mean asthma symptom scores evaluated using the ACT showed good improvement in both medium- and high-dose FF-UMEC-VI, accompanied by FEV1 improvement, and good control was achieved 4 weeks after switching to FF-UMEC-VI in the present study. Thus, when patients with asthma on ICS-LABA therapy have residual asthma symptoms, switching to FF-UMEC-VI contributes to an improvement in asthma symptoms and FEV1, regardless of the FeNO level. Similarly, in the CAPTAIN study, improvement in the asthma control questionnaire-7 (ACQ-7) score was seen as early as week 4 and was sustained until week 24 (4).

Considering the change in ACT score, 92% of the T100 patients changed from <20 at baseline to ≥20 (good + total) after switching to a medium dose of FF-UMEC-VI, and 65% of the T200 patients achieved a score of ≥20 using a high dose of FF-UMEC-VI. Therefore, a high dose of FF-UMEC-VI is also a treatment option that should be considered before oral corticosteroid therapy or biologics induction. Consistent with these findings, a recent retrospective study analyzed a database in the USA and reported that FF-UMEC-VI resulted in significantly reduced oral corticosteroid use, SABA use, and asthma-related exacerbations in patients with asthma, compared with the period prior to treatment (12).

Despite these favorable results, the present study showed that 35% of the patients still had poor control after switching to a high dose of FF-UMEC-VI. Similarly, 36% of the patients were non-responders to a high dose of FF-UMEC-VI in the CAPTAIN study (4). Patients who did not improve after switching to high-dose FF-UMEC-VI were characterized by low baseline FEV1, low ACT score and OCS use at baseline. Such patients may require other treatment options.

In the present study, the cough VAS significantly decreased but did not reach the MCID. In a retrospective study of patients prescribed FF-UMEC-VI as the first-line therapy, cough VAS reached the MCID as early as week 2 after the initiation of FF-UMEC-VI (24). Cough VAS improvement may not have reached the MCID in the present study because FF-UMEC-VI was not used as the initial therapy, and coughing may have already been repressed to some degree before switching to FF-UMEC-VI. A previous study showed that an initial treatment of ICS-LABA improved coughing (25). To verify the effect of FF-UMEC-VI on coughing, it is necessary to employ other coughing assessment methods in addition to the VAS.

This study evaluated whether the initial inhalation instruction was effective at 3 months later. Inadequate inhalation techniques can cause asthma treatment failure. It is recommended that health care providers educate patients about inhalation techniques and repeat checks to ensure correct use of inhalers (8). The rate of patients with a history of asthma who were performing inhalation correctly 28 days after receiving initial inhalation training on the Ellipta® device was 96% to 98%. Patients in that study were 70% female, with an average age of 50.9 years (26). In present study, average age was similar to that study (26) and the number of errors made while performing the inhalation technique was almost none in all enrolled patients, further demonstrating the effectiveness of the Ellipta® device. However, a number of unique physical and cognitive issues exist in elderly individuals that may increase the risk of misuse of inhaler (27). The DASC-8 is used to assess cognition, and an evaluation of the relationship between the DASC-8 and the number of errors made during inhalation was attempted, but the relationship could not be evaluated due to the young patients who were included, resulting in a small number of DASC-8-positive (score of >8) patients. It would be helpful to clarify the relationship between inhalation technique acquisition and cognition regarding the Ellipta® device for elderly patients with asthma in the future.

There were no serious side effects and hoarseness was the most common. No patients experienced asthma exacerbation during the study period.

## 4 Study limitations

There was no control group because the present study was limited to real-world data, and the population size was small. Although the sample size for this study was calculated based on a two-group comparison, further validation in a larger number of patients is needed. Furthermore, the peripheral blood eosinophil count was not included in the evaluation because blood sampling is invasive, and the number of eosinophils could not be obtained quickly. In daily clinical practice, when determining whether a change in inhaler medication is necessary, the measurement of FeNO is noninvasive and can be obtained in real time; however, measuring the eosinophil count is more difficult. In this study, the work to calculate a correction equation to compensate for measurement differences between the spirometer or FeNO measurement instruments was not performed. We could not find any studies that corrected the measurement differences of the instruments used in this study. It cannot be ruled out that use of spirometer and FeNO measurement instruments in each site might have affected the measured values.

In an earlier report, patients who did not respond to short-term (6 weeks) ICS treatment had no change in asthma control when inhaled steroids were continued for up to 16 weeks (28). Therefore, a 12-week evaluation in the present study period was acceptable with regard to evaluate the effect of switching the ICS composition. However, further evaluation of asthma control with triple therapy may need to evaluate longer term effects. Some patients did not come to the clinic or hospital every 4 weeks, resulting in missing data, but statistical methods were used to compensate for this. Finally, we required patients to bring the FF-UMEC-VI device for every visit to check its counter for monitoring adherence, but many patients did not bring their inhalers with them. To see the effect of a single device, it should be evaluated in a crossover study while monitoring adherence in the future study.

## 5 Conclusions

This real-world study showed that switching therapy from ICS-LABA to FF-UMEC-VI was effective in improving FEV1 and asthma symptoms, regardless of the FeNO level.

## Data Availability

The raw data supporting the conclusions of this article will be made available by the authors, without undue reservation.
