# Correction: Real-life effectiveness of once-daily single-inhaler triple therapy (FF-UMEC-VI) after switching from dual therapy (ICS-LABA) in patients with symptomatic asthma: trelegy ellipta for real asthma control study

**DOI:** 10.3389/falgy.2025.1683768

**Published:** 2025-09-23

**Authors:** Yoshitomo Kushima, Yasuo Shimizu, Ryo Arai, Kazuyuki Chibana, Yuka Shimizu, Masahiro Amagai, Akihiro Takemasa, Naoya Ikeda, Meitetsu Masawa, Atsushi Kushima, Hiroaki Okutomi, Yusuke Nakamura, Rinna Tei, Yuki Ando, Nana Yazawa, Yuto Goto, Yasuo Haruyama, Tatsuo Yukawa, Seiji Niho

**Affiliations:** 1 Department of Pulmonary Medicine and Clinical Immunology, Dokkyo Medical University, Mibu, Tochigi, Japan; 2 Kushima Internal Medicine Clinic, Moka, Tochigi, Japan; 3 Amagai Internal Medicine Clinic, Tochigi, Tochigi, Japan; 4 Integrated Research Faculty for Advanced Medical Sciences, Dokkyo Medical University, Tochigi, Japan; 5 Yukawa Clinic of Internal Medicine, Utsunomiya, Tochigi, Japan

**Keywords:** asthma, cognition, fluticasone furoate, forced expiratory flow volume, trelegy, triple therapy, umeclidinium, vilanterol

In the published article, there was an error. There was an incorrect description of statistical methods.

A correction has been made to section **1.4 Statistical analysis**, paragraph number 2. This sentence previously stated:

“Changes over time between groups were evaluated by repeated measures, and comparisons of the parameters between the baseline and each time point were evaluated using the Bonferroni method.”

The corrected sentence appears below:

“Changes over time between groups were evaluated by repeated measures, and comparisons of the parameters between the baseline and each time point were evaluated using the Least significant difference (LSD) method.”

In the published article, there was an error in the legend for Figure 2 as published. There was an incorrect description of statistical methods.

The sentence previously stated:

“The bars represent the 95% CI, *p*-values are for repeated measurements within a group, and the Bonferroni method was used for time comparisons.”

The corrected sentence appears below:

“The bars represent the 95% CI, *p*-values are for repeated measurements within a group, and the Least significant difference (LSD) method was used for time comparisons.”

In the published article, there was an error in Figure 3C as published. There was an incorrect description of the ratio for patients in the right-side pie chart for the value under “Well *n* = 10”. The value 10% was corrected to 48%. The corrected figure appears below.

**Figure 3:**
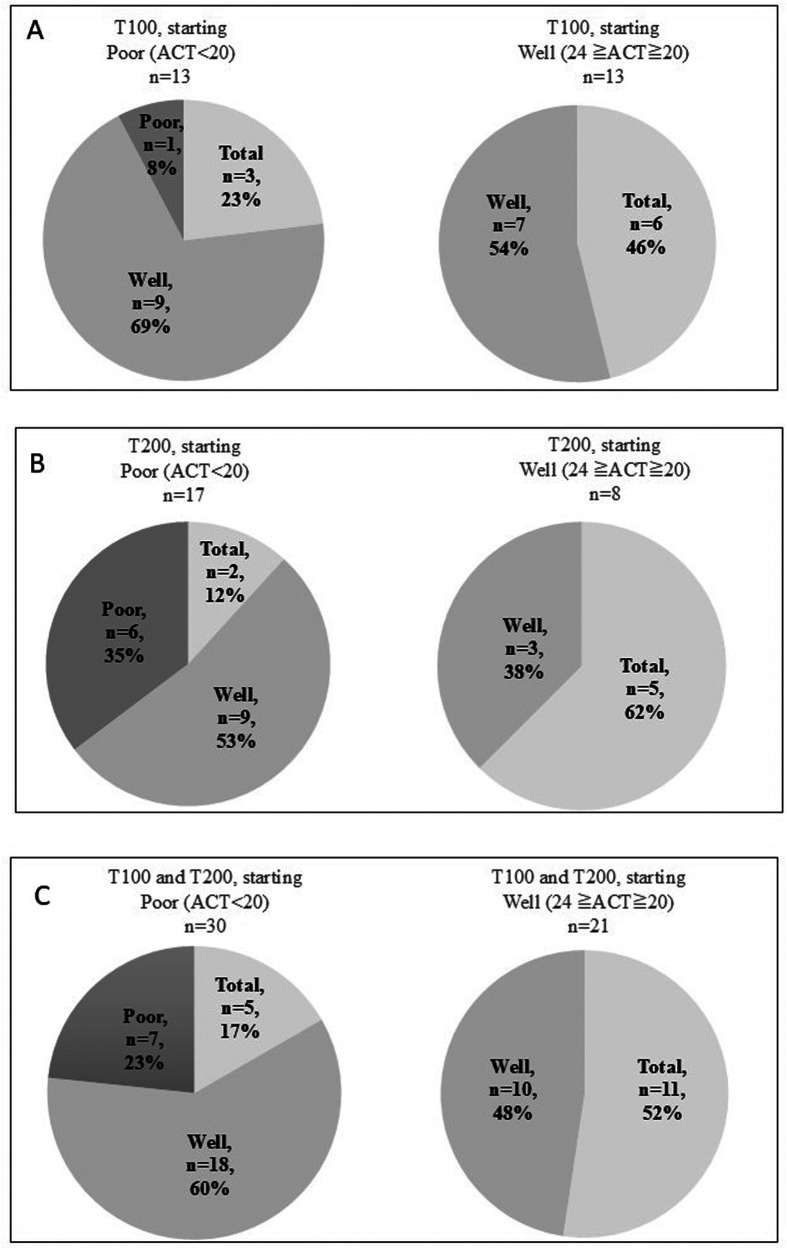
ACT scores and the changes in patients after switching to 100–62.5-25 µg of FF-UMEC-VI (T100 group) **(A)**, 200–62.5-25 µg of FF-UMEC-VI (T200 group) **(B)** Combined T100 and T200 group data for ACT scores before and after switching to FF-UMEC-VI **(C)** Patients with ACT scores of <20 before starting FF-UMEC-VI were defined as having poor control, patients with ACT scores of ≥20 and ≤24 before starting FF-UMEC-VI were defined as having good control, and a score of 25 was defined as total control. FF-UMEC-VI, fluticasone furoate-umeclidinium-vilanterol; ACT, asthma control test.

The original article has been updated.

